# Exploring adverse childhood experiences (ACEs) among Ugandan university students: its associations with academic performance, depression, and suicidal ideations

**DOI:** 10.1186/s40359-023-01044-2

**Published:** 2023-01-13

**Authors:** Moses Muwanguzi, Mark Mohan Kaggwa, Sarah Maria Najjuka, Mohammed A. Mamun, Innocent Arinaitwe, Jonathan Kajjimu, Elicana Nduhuura, Scholastic Ashaba

**Affiliations:** 1 Faculty of Medicine, Mbarara University of Science and Technology, Mbarara, Uganda; 2 Department of Psychiatry, Mbarara University of Science and Technology, Mbarara, Uganda; 3 Department of Psychiatry and Behavioural Neurosciences, McMaster University, Hamilton, ON Canada; 4 Makerere University, College of Health Sciences, Kampala, Uganda; 5 CHINTA Research Bangladesh, Savar, Dhaka Bangladesh; 6 Department of Public Health and Informatics, Jahangirnagar University, Savar, Dhaka Bangladesh

**Keywords:** Adverse childhood experiences, Childhood trauma, Depression, Suicidal ideations, Academic performance, University students

## Abstract

**Background:**

Adverse childhood experiences (ACEs) among university students have been linked to a variety of factors and have been shown to have a dose–response relationship with adult health and behavior.

**Objective:**

To investigate the effect of exposure to ACEs on academic performance, depression, and suicidal ideations among university students.

**Methods:**

A cross-sectional survey among university students at a public university in southwestern Uganda was conducted in 2021, integrating the Adverse Childhood Experiences International Questionnaire for assessing ACEs, the Patient Health Questionnaire for assessing depression symptoms and suicidal ideations, and questions assessing the family structure and academic performance as adopted from similar studies. Regression analysis was performed, and 3 models were generated to answer the study hypotheses.

**Results:**

A total of 653 undergraduate university students with a mean age of 22.80 (± 3.16) years were recruited. Almost all students (99.8%) experienced one or more ACEs, with physical abuse being the common ACE reported. The average depression symptom severity was statistically higher among individuals who experienced any form of ACEs. No relationship was observed between the ACEs experienced and self-rated academic performance. Similarly, on regression analysis, the cumulative number of ACEs was not associated with self-rated academic performance (β =  − 0.007; 95% CI − 0.031 to 0.016; *p* = 0.558). However, the cumulative number of ACEs was positively associated with depression symptom severity (β = 0.684; 95% CI 0.531–0.837; *p* < 0.001), as well as increased the likelihood of suicidal ideations (aOR = 1.264; 95% CI 01.090–1.465; *p* < 0.001).

**Conclusions:**

The burden of ACEs is exceedingly high among Ugandan university students, highlighting the urgency in strengthening effective child protection strategies to protect Uganda’s rapidly growing population from mental ill-health and avoid future psychological disability, a burden to the healthcare system. The study's findings will also be useful to practitioners/policymakers working to prevent/limit child maltreatment globally.

## Introduction

### Overview of the impacts of ACEs

Childhood is a period of immense cognitive, behavioral, physical, and emotional development, hence regarded as potentially a period of vulnerability [1]. Therefore, for optimal development, children need a safe and supportive environment free from violence [1]. Child abuse (psychological, sexual, physical), neglect (emotional, physical), and household dysfunction (violence against mother, living with household members who were substance abusers, mentally ill or suicidal, ever imprisoned) are all forms of adverse childhood experiences (ACEs) [2, 3]. Exposure to ACEs results from a complex interplay between multiple predisposing and protective factors (like support from a trusted adult) [4], which appear in the individual, social, community, and societal contexts [5].

The most common forms of ACEs reported in African countries are physical abuse, household dysfunction, and emotional abuse [6–8]. Studies among youths-predominant societies in Africa reported diverse prevalence rates of experiencing at least one ACE, ranging from 46.2% among Nigerian adults [9] to 90% among rural Ugandan adults [8, 10]. Among university and college students, the prevalence rates of experiencing at least one ACE ranged from 58.3% from a Zambian college [11] to 97.6% among Tanzanian pre-college students [12], which shows a slightly higher burden of childhood maltreatment in East African student communities. In addition, studies have shown that ACE survivors have higher risks for mental health problems and behavioral and emotional dysregulation resulting in long-term dysfunction in adulthood [3, 8–10].

Exposure to traumatic events is associated changes in the brain. For example, it may trigger a fight–flight/freeze response that floods the brain with corticotropin-releasing hormone, a normal and protective response in stressful situations [13]. However, repeated exposure to continuous corticotropin-releasing hormone production by the brain may result in a permanently heightened state of alertness in the child and the inability to return to the recovered state, causing chronic stress [14]. This impairs executive functions, memory, and attention deficits due to the constantly heightened neurological state which may later impair the execution of academic tasks [14]. These pathophysiologic disruptions have been found to be associated with depression symptoms and suicidality following early life stress [14, 15]. In addition, life transitions of emerging adulthood (aged 18–29 years—as among university students) may generate stress and psychological distress, which may compound on the existing childhood trauma resulting in poorer self-rated health and life satisfaction, depressive symptoms, anxiety, illicit drug use, excessive alcoholism, mental illness, and risky sexual behavior [4, 12, 16].

Amongst the scarcity of literature about ACEs and their impact on young adults’ lives in Uganda, studies have shown a significant impact of ACEs on adult life [10, 17]. For example, the cumulative number of ACEs reported had a strong positive association with depression symptom severity and suicidal ideation as well as heavy alcohol consumption among a younger population of emerging adults in the Southwestern Uganda [10]. In addition, ACE exposure has been associated with early initiation of alcohol consumption among youths as well as heavy alcohol consumption behaviors among adults in Uganda [8, 17] which threatens their mental health. These patterns of ACEs and adulthood complications of ACEs have been observed in other parts of sub-Sahara Africa (SSA)—a region with similar cultural practices as Uganda. For example, in South Africa, a study reported high ACEs exposure to increase the likelihood of having preterm births and perinatal alcohol and tobacco abuse [18]. Another study in four countries in SSA including Uganda reported an association between ACEs and self-reported drunkenness among adolescents [7]. Childhood trauma was also documented to be associated with depression and suicidality among adolescents living with HIV in Uganda [19]. In addition, a systematic review from developed countries reports a wide range of adverse outcomes of ACEs exposure to emerging adults, including problematic drug use and interpersonal and self-directed violence, smoking, heavy alcohol use, poor self-rated health, cancer, heart disease, and respiratory disease, physical inactivity, overweight or obesity, and diabetes mellitus [20].

**Fig. 1:**
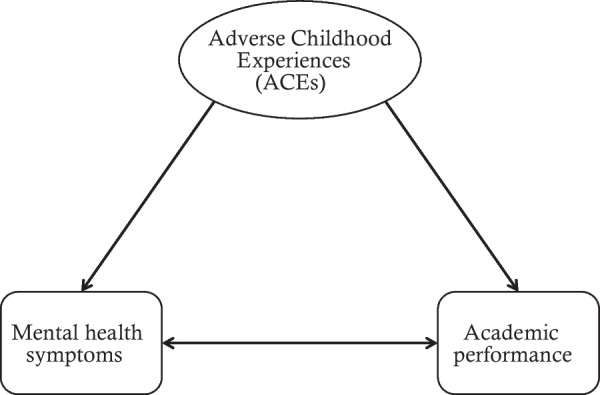
The proposed theoretical framework

With a proposed theoretical framework (Fig. 1), we conducted a review of existing literature on ACEs, mental health symptoms and academic performance among university students to understand better how these constructs interact in the setting of emerging adulthood/university students.

### Adverse childhood experiences among university students

Although the burden of ACEs varies globally according to the type of ACEs reported and the context/setting; the pooled prevalence of ACEs based on a global systematic review among school-aged youth reported that almost two-thirds of youths experience ACEs [21]. Over three-quarters, 76.2% of young adults from 8 universities in Vietnam reported exposure to at least 1 ACE, and 21% reported exposure to 4 or more ACEs [16]. This high exposure to ACEs among countries with a competent legal system may predict unacceptably high prevalence rates in developing and low-income countries such as Uganda with insufficient child protection systems [22]. Moreover, in Uganda’s immediate neighbor, Tanzania, the burden of ACEs exposure among pre-university students is even higher than that among university students [12]. Therefore, ACEs among university students represent a spill-over of resilient ACEs victims who may be at risk of health problems in a more stressful university environment.

### Adverse childhood experiences and resultant academic performance

Based on a literature review in 2014, students with maltreatment histories are likely to experience difficulties across a broad spectrum of functional domains, including educational attainment [23]. The findings have also been reported in a recent study among 404 American university students, where a negative relationship between post-secondary academic performance with the cumulative number of ACEs was reported [24]. Such findings can be explained by an animal model which demonstrates the neurobiology of single prolonged stress, that is, chronic stress impairs the neurodevelopment of the hippocampus, medial prefrontal cortex, limbic system, and amygdala [25], which are parts of the brain responsible for memory, thinking, and learning, hence the impairment in school performance. As reported among other populations, childhood trauma among university students is associated with a diverse array of mental health problems, which have been associated with poor academic outcomes [23, 26].

### Relationships between ACEs, depression and suicide

Most studies among college and university students have reported depression, suicidality, anxiety, and post-traumatic stress disorder, as the most typical mental illnesses associated with a history of exposure to ACEs [27–30]. A study among South African youths found that girls who experienced emotional neglect were about two times and five times more likely to develop depression and suicidality respectively, whereas their male counterparts were three times more likely to develop depression [31]. In Uganda, two studies were done among school-going adolescents and young adults to explore a negative relationship between the severity of depression and the loss of a parent and family alcohol consumption [32]. Whereas there is a positive association between the cumulative number of ACEs and mental health symptom severity [8, 10], scanty literature still exists regarding university students.

### Academic performance and mental health

Depression symptomatology and academic achievement among university and college students have existed in a bidirectional inverse relationship as reported by researchers in high- and low-income countries [33–37]. Results from a study among Australian high school students reported that poor academic performance as a trigger of depressive symptoms [36], whereas others have shown that the depressive symptoms and suicidal behavior reported among college and university students are negatively correlated with academic grades [33, 34]. Among South Australian high school students, those who perceived their academic performance as above average scored better on measures of self-esteem, depressive symptoms, locus of control, and suicidality assessment [36], which confirms the negative associations.

As evidenced from the above literature, earlier research in Uganda has demonstrated the detrimental effects of ACE-exposure on mental health and academics, but little is known about the same in Ugandan university students, yet the main hope of a nation lies in the education of its youth. To note, majority of the population are below the age of 25 years [38].

### The present study

After adjusting for sociodemographics and family environment-related variables, this study’s research objective was to investigate the effect of ACEs exposure on academic performance and mental health among university students. Based on our review of the literature, we advanced the following hypotheses, which the study will test.

#### Hypothesis 1

Exposure to ACEs is associated with self-rated academic performance of university students.

#### Hypothesis 2

Exposure to ACEs is associated with current depression among university students.

#### Hypothesis 3

Exposure to ACEs is associated with suicidal ideation among university students.

## Methods

### Study design and setting

The present cross-sectional study collected data through an online survey method. The survey was carried out using a Google Form from April 3 to May 23, 2021. Students enrolled in a public university, Mbarara University of Science and Technology (MUST), in southwestern Uganda were eligible to participate in this survey.

### Study sample size estimation

MUST had over 4269 undergraduate students enrolled (as of 2019) in its 6 faculties [i.e., Faculty of Medicine (n = 1674), Faculty of Computing and Informatics (n = 339), Faculty of Business and Management Sciences (n = 964), Faculty of Science (n = 550), Faculty of Applied Sciences and Technology (n = 481), and Faculty of Interdisciplinary Studies (n = 261)], as provided by the university administration. All undergraduate students during the 2019/2020 academic year were eligible for this study, and the minimum sample size to produce a statistical power of 80% was calculated using Epi Info StatCalc for population surveys version 7.2.2.6. Using a population size of 4269 undergraduates, the expected frequency of ACEs at 50% (*since no similar study had been conducted among university students in Uganda*), an acceptable margin of error of 5%, and a design effect of 1.0, the minimum calculated sample size was 352. Both convenience sampling via online platforms and snowball sampling were used to recruit the students in the study.

### Data collection and procedure

To ensure effective communication between students and the university leadership, each student has an institutional email address for official communications, and these emails were used to collect data for the present study. In addition, students also have different social media platforms including WhatsApp groups, Facebook groups, and other socializing groups, where the online survey link was shared for data collection purpose. The research team circulated the online survey link within the faculty platforms and student social media networks like WhatsApp groups and emails. The participants were further requested to circulate the link to their networks within the same institution.

### Study measures

The online survey tool included a consent form, where informed consent was first obtained before participating in this study. The questionnaire consisted of sections capturing information on (1) socio-demographics, (2) family environment, (3) academic performance, (4) mental health outcomes (depression and suicidal ideation), and (5) adverse childhood experiences.

#### Socio-demographics

The questionnaire collected information on the socio-demographic characteristics including age (in completed years), gender, marital status (single, married/cohabiting, and separated/divorced), and the region of the country of origin (Central, Western, Eastern, and Northern).

#### Family environment

Descriptive information about the family environment was collected that included the participant’s family type (nuclear family, extended family, step-parent family, grandparent family, single-parent family), primary care provider/sponsor for paying university tuition fees (parent, step-parent, uncle/aunt, sibling, guardian, grandparent, Non-Government Organisation (NGO), self-sponsored), number of family members, number of children, and birth position. Due to non-normality in the number of family and children distribution, we re-categorized these two continuous variables into ordinal categories utilizing the four quartiles. Participants were also asked about the highest educational level attained by their parents (whether alive or deceased).

#### Academic performance

Participants were asked to self-rate their ‘overall academic performance’ on a 5-point Likert question (1 = very poor, 2 = poor, 3 = good, 4 = very good, and 5 = excellent), recorded as a continuous variable. Their Cumulative Grade Point Average (CGPA) was assessed under the default categories [i.e., CGPA of 4.40–5.00 (first class), 3.60–4.39 (second class-upper), 2.80–3.59 (second class-lower), and 2.00–2.79 (third class)] as recommended by the Uganda National Council for Higher Education [39]. The categorization given by the Uganda National Council for Higher Education was adopted to avoid social desirability bias in reporting the discrete individual CGPA. In addition, this study asked for other academic information such as the year of study and the faculty of the study.

#### Mental health outcomes

Depressive symptoms were assessed by the Patient Health Questionnaire (PHQ-9). The PHQ-9 is a brief, easily administered, and scored screening questionnaire that is used to screen for depression [40–42]. Responses to the nine items were recorded based on a 4-point Likert scale (0 = not at all, to 3 = nearly every day), and its total score ranges from 0 to 27. A score of 10 is used as the cutoff score for having depression, or not, which has shown 88% sensitivity and 88% specificity for the depression [43]. Depression was presented both as a binary categorical variable (having depression or not), and as a continuous variable (depression symptoms severity). The PHQ-9 scale has previously been used among Ugandan university students [44, 45]. The tool showed good reliability among students and different cultures in the country [42, 46–48]. The Cronbach alpha of the PHQ-9 in the present study was 0.81.

The ninth item from the PHQ-9, “*Over the last two weeks, how often have you been bothered by thoughts that you would be better off dead or hurting yourself in some way*”, was used to assess suicidal ideation. Participants were categorized as suicidal ideators if they had a response to this question of “several days (1)”, “nearly every day (2)” and “more than half of the days (3)”. Those who scored ‘0’ were classified as having no suicidal ideation, whereas a score of at least 1 was used for the suicidal ideation cutoff point. Suicidal ideation was presented as a binary variable (having suicidal ideation or not).

#### Adverse childhood experiences

Adverse Childhood Experiences were assessed both by categories and by the number of adversities experienced. The ACE-IQ is designed to be integrated within broader health surveys to analyze associations between adverse childhood experiences and subsequent health outcomes and health risk behaviors. This study predicts exposure to ACEs on account of the current socio-health status using the standardized WHO-developed Adverse Childhood Experiences International Questionnaire (ACE-IQ) [49]. The ACE-IQ tool measures thirteen categories of ACEs, which were all assessed in this study. The ACE-IQ consists of 29 questions assessing the 13 childhood adversities. Each adversity has a binary response where any event experience is categorized as 1, and no experience of the event is categorized as 0. The total ACE scores ranged from 0 to 13, where the higher score represents the higher number of childhood adversities experienced by a participant. The tool has been applied to the African context and found adequate in assessing exposure to ACEs among individuals in low- and middle-income countries [50–52]. In an ACEs study in Kenya, the ACE-IQ tool was used with a reported good exposure assessment in a similar population age group (18–40 years) [53]. The tool had a Cronbach alpha of 0.82 in this study.

### Ethical consideration

The study was conducted in accordance with the Declaration of Helsinki [54]. This study's formal ethical approval was obtained from the Research Ethics Committee at the Mbarara University of Science and Technology (MUSTREC#04/01-21). Informed consent was obtained from all study participants. Participants were informed about the nature of the questions, especially ACE-IQ, since some questions would potentially raise negative emotions about their previous childhood adverse events. In case of distress (based on participants’ reports), the study provided psychological management by linking them to a psychiatric team through a link-to-psychological help that was integrated into the online tool.

### Statistical analysis

A Google spreadsheet with the collected data was imported into, cleaned, and analyzed using the statistical software, STATA V.15. Descriptive statistics (e.g., percentage and frequency) and (mean & standard deviation) were used to present categorical and continuous data, respectively. For continuous variables, normal distribution was assumed if kurtosis was below 7, and the skewness was below 2 [55]. In addition, the Gaussian assumption was also used to assess normality based on the Shapiro-Wilks test and histograms. The analysis of variance (ANOVA) test and student *t*-test were performed to determine the statistical difference between the number of ACEs reported across the different study independent variables. The main outcome variables in this study were self-rated academic performance, depression symptoms severity, and the presence of suicidal ideation. To determine the relationships between exposure to each type of ACEs and outcome variables, means and standard deviation for normally distributed continuous outcome variables (self-rated academic performance and depression symptoms severity) and Chi-square tests for suicidal ideations were computed. Multi-collinearity test was done and all independent variables had a Variance Inflation Factor (VIF) less than 3. Hence, regression analysis was conducted utilizing the cumulative number of ACEs as the main predictor variable, and three models were generated (model 1 for self-rated academic performance, model 2 for depression symptom severity, and model 3 for suicidal ideation). Multiple linear regression was run for model 1 and 2, whereas adjusted logistic regression was done for model 3. A *p* value < 0.05 was set as statistically significant with a 95% of the confidence interval.

## Results

### Characteristics of the participants

In the total sample (N = 653), the participant’s mean age was 22.8 (± 3.16) years, with the majority being males (65.2%). Up to half of the students came from the nuclear family type (54.1%), in which 4 in every five students (81.2%) were primarily cared for by their parents. The majority of the participants (45.9%) reported their academic performance as Second Class-Upper. The majority were in their 2nd year of study (34.0%), and two-fifth (41.5%) belonged to the Faculty of Medicine. Mental health assessment revealed that almost a third (28.2%) of the students were depressed, and of which 7.0% had suicidal ideation (Table 1).

**Table 1:** Distribution between studied variables and adverse childhood experiences

Study variables	Total sample; n, %	ACEs exposure to		
		Mean ± SD	F/t-test	*p* value
*Socio-demographic variables*				
Age (in completed years)		22.80 ± 3.16	2.00	0.005
Gender				
Female	227, 34.8%	6.32 ± 2.60	1.37	0.172
Male	426, 65.2%	6.02 ± 2.69		
Marital status				
Single	601, 92.0%	6.10 ± 2.65	0.25	0.778
Married/cohabiting	44, 6.7%	6.30 ± 2.75		
Separated/divorced	8, 1.2%	6.63 ± 2.88		
Region of residence				
Central	166, 25.4%	6.27 ± 2.52	0.67	0.571
Eastern	114, 17.5%	6.22 ± 2.84		
Northern	41, 6.3%	6.39 ± 1.83		
Western	332, 50.8%	5.98 ± 2.75		
*Family environment*				
Type of family				
Extended family	205, 31.4%	6.35 ± 2.50	10.68	< 0.001
Grand parent family	15, 2.3%	7.53 ± 3.07		
Nuclear family	353, 54.1%	5.64 ± 2.64		
Single parent family	65, 10.0%	7.05 ± 2.51		
Step family	15, 2.3%	8.80 ± 2.18		
Primary care provider/Sponsor for paying university tuition fees				
Parent	530, 81.2%	5.98 ± 2.55	5.09	< 0.001
Step parent	3, 0.5%	8.67 ± 2.31		
Uncle/aunt	33, 5.1%	6.06 ± 2.30		
Sibling	15, 2.3%	6.53 ± 2.29		
Guardian	30, 4.6%	6.83 ± 3.12		
Grandparent	15, 2.3%	9.60 ± 2.90		
NGO	19, 2.9%	6.00 ± 3.07		
Self-sponsored	8, 1.2%	4.88 ± 3.76		
Number of family members				
0–6	239, 36.6%	6.05 ± 2.72	2.12	0.097
7–8	177, 27.1%	5.99 ± 2.50		
9–10	117, 17.9%	5.92 ± 2.69		
11–25	120, 18.4%	6.67 ± 2.71		
Number of children in family				
0–4	196, 30.0%	6.01 ± 2.67	0.75	0.523
5–6	212, 32.5%	6.22 ± 2.47		
7–8	127, 19.5%	5.92 ± 2.53		
9–18	118, 18.0%	6.36 ± 3.09		
Participant’s birth position				
1st	143, 21.9%	6.10 ± 2.69	0.36	0.780
2nd	151, 23.1%	6.27 ± 2.61		
3rd	144, 22.1%	5.95 ± 2.58		
4th and more	215, 32.9%	6.14 ± 2.74		
Mother’s education level				
None	110, 16.8%	6.55 ± 2.69	2.82	0.038
Primary	146, 22.4%	6.23 ± 2.70		
Secondary	163, 25.0%	5.65 ± 2.72		
Tertiary	234, 35.8%	6.18 ± 2.55		
Father’s education level				
None	89, 13.6%	7.07 ± 2.71	7.85	< 0.001
Primary	95, 14.6%	6.23 ± 2.62		
Secondary	154, 23.6%	5.41 ± 2.73		
Tertiary	315, 48.2%	6.17 ± 2.53		
*Academic performance*				
CGPA grading				
First class	84, 12.9%	6.02 ± 3.13	2.61	0.035
Second class—upper	300, 45.9%	6.03 ± 2.44		
Second class—lower	162, 24.8%	6.61 ± 2.82		
Third class	14, 2.1%	4.79 ± 2.64		
Preferred not reporting	87, 14.2%	5.87 ± 2.49		
Year of study				
1st	171, 26.2%	6.05 ± 2.83	3.89	0.004
2nd	222, 34.0%	6.27 ± 2.64		
3rd	106, 16.2%	6.66 ± 2.54		
4th	94, 14.4%	5.96 ± 2.39		
5th	60, 9.2%	5.05 ± 2.57		
Faculty of study				
Applied Sciences and Technology	65, 10.0%	5.94 ± 2.78	3.72	0.003
Business and Management Studies	88, 13.5%	5.28 ± 2.73		
Computing & Informatics	69, 10.6%	6.64 ± 3.17		
Interdisciplinary Studies	9, 1.4%	4.78 ± 1.56		
Medicine	271, 41.5%	6.10 ± 2.38		
Science	151, 23.1%	6.57 ± 2.71		
*Mental health outcomes*				
Depression (cutoff 10)				
Yes	184, 28.2%	7.32 ± 2.79	7.51	< 0.001
No	469, 71.8%	5.65 ± 2.45		
Suicidal ideations				
Yes	46, 7.0%	7.04 ± 3.14	2.45	0.015
No	607, 93.0%	6.05 ± 2.61		

*ACE(s)* adverse childhood experience(s), *SD* standard deviation, *NGO* Non-Government Organization, *CGPA* Cumulative Grade Point Average

### Relationships between the cumulative number of ACEs and the studied variables

Sociodemographics

Table 1 reports the distribution between the number of ACEs exposures and the studied variables. Participants’ age showed a significant relationship with the cumulative number of ACEs (22.80 ± 3.16; *t* = 2.00, *p* = 0.005). It is noted that females and separated/divorced participants reported experiencing more ACEs compared to their counterparts, (6.32 ± 2.60; F = 1.37, *p* = 0.172) and (6.63 ± 2.88; F = 0.25, *p* = 0.778), respectively, although not statistically significant.

#### Family environment

The relationship of type of family regarding ACEs was statistically significant, where participants from stepfamily and grand-parent families had reported higher exposure to ACEs than other types (F = 10.68, *p* < 0.001). Homogeneously, participants with step-parents and grandparents as primary care providers reported more ACEs (F = 5.09, *p* < 0.001). In addition, a lower education level of both parents (mother and father) was associated with reporting more ACEs (Table 1).

#### Academic performance

There was a statistically significant difference between academic performance by CGPA and the faculty of the study with the reported number of ACEs reported (F = 2.61, *p* = 0.035) and (F = 3.72, *p* = 0.003), respectively. Moreover, lower ACEs were reported among students with higher academic years (F = 3.89, *p* = 0.004) (Table 1).

#### Mental health outcomes

In respect to mental health problems, those participants who are currently depressed (7.32 ± 2.79 vs. 5.65 ± 2.45, t = 7.51, *p* < 0.001), and have suicidal ideations (7.04 ± 3.14 vs. 6.05 ± 2.61; t = 2.45, *p* = 0.015) reported experiencing higher ACE compared to those who were not (Table 1).

### Distribution of the number of adverse childhood experiences

**Fig. 2:**
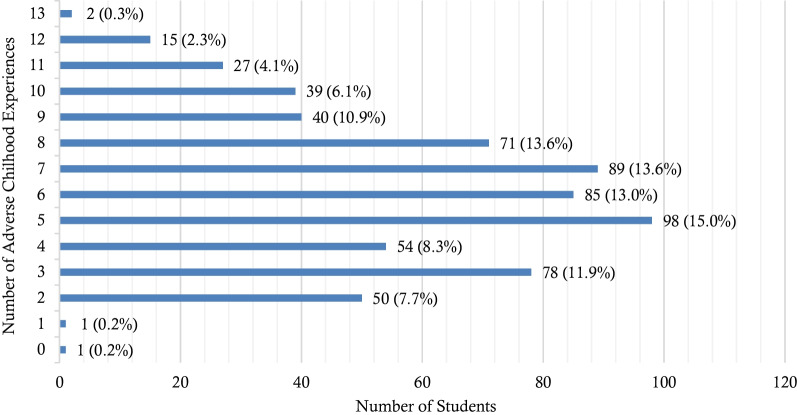
Prevalence of exposure to adverse childhood experiences, by the cumulative number of ACEs

The total number of ACEs experienced ranged from 0 to 13 adversities; most participants experienced five ACEs (15%). The mean number of ACEs was 6.1 (± 2.7), with a median of six, and an interquartile adversity range of 11 (see Fig. 2).

### Relationships between exposure to different types of ACEs and studied predictor variables

Table 2 shows the associations between exposure to each type of ACEs and the outcome variables. Almost all participants (99.9%) reported having experienced at least one ACE, with the most prevalent type of ACEs being physical abuse (99.8%). In addition, nearly a third (29.1%) of all participants were sexually abused. Also, over two-thirds (68.0%) of the participants reported having experienced emotional neglect, whereas more than half (57.3%) were subjected to actual emotional abuse.

**Table 2:** Relationships between the types of ACEs and the mental health outcomes and self-rated academic performance

ACE	Exposure (n, %)	Mental health outcomes					Self-rated academic performance	
		Depression		Suicidal ideations				
ACEs types		Mean ± SD	*p* value	Yes (n, %)	No (n, %)	*p* value	Mean ± SD	*p* value
Physical abuse								
Yes	652, 99.8%	6.72 ± 5.52	N/A	46, 7.1%	606, 92.9%	0.783	3.80 ± 0.87	N/A
No	1, 0.2%	2		0, 0%	1, 100%		3	
Emotional abuse								
Yes	374, 57.3%	7.69 ± 5.62	< 0.001	25, 6.7%	349, 93.3%	0.677	3.79 ± 0.84	0.678
No	279, 42.7%	5.41 ± 5.10		21, 7.5%	258, 92.5%		3.82 ± 0.90	
Contact sexual abuse								
Yes	190, 29.1%	8.58 ± 6.03	< 0.001	25, 13.2%	165, 86.8%	< 0.001	3.77 ± 0.92	0.539
No	463, 70.9%	5.95 ± 5.11		21, 4.5%	442, 95.5%		3.81 ± 0.84	
Alcohol and/or drug abuser in the household								
Yes	180, 27.6%	7.91 ± 6.00	0.001	12, 6.7%	168, 93.3%	0.816	3.73 ± 0.82	0.183
No	473, 72.4%	6.26 ± 5.26		34, 7.2%	439, 92.8%		3.83 ± 0.88	
Incarcerated household member								
Yes	132, 20.2%	8.82 ± 5.84	< 0.001	15, 11.4%	117, 88.6%	0.030	3.76 ± 0.82	0.519
No	521, 79.8%	6.18 ± 5.31		31, 5.9%	490, 94.1%		3.81 ± 0.88	
Someone chronically depressed, mentally ill, institutionalized or suicidal								
Yes	111, 17.0%	8.63 ± 6.28	0.001	12, 10.8%	99, 89.2%	0.089	3.72 ± 0.84	0.284
No	542, 83.0%	6.33 ± 5.27		34, 6.3%	508, 93.7%		3.82 ± 0.86	
Household member treated violently								
Yes	433, 66.3%	7.73 ± 5.58	< 0.001	32, 7.4%	401, 92.6%	0.628	3.77 ± 0.85	0.157
No	220, 33.7%	4.72 ± 4.82		14, 6.4%	206, 93.6%		3.87 ± 0.89	
One or no parents, parental separation or divorce								
Yes	301, 46.1%	7.99 ± 5.58	< 0.001	25, 8.3%	276, 91.7%	0.244	3.76 ± 0.89	0.236
No	352, 53.9%	5.63 ± 5.23		21, 6.0%	331, 94.0%		3.84 ± 0.84	
Emotional neglect								
Yes	444, 68.0%	7.31 ± 5.69	< 0.001	35, 7.9%	409, 92.1%	0.222	3.77 ± 0.87	0.261
No	209, 32.0%	5.45 ± 4.91		11, 5.3%	198, 94.7%		3.86 ± 0.85	
Physical neglect								
Yes	163, 25.0%	8.33 ± 5.88	< 0.001	13, 8.0%	150, 92.0%	0.592	3.82 ± 0.90	0.798
No	490, 75.0%	6.18 ± 5.29		33, 6.7%	457, 93.3%		3.79 ± 0.86	
Bullying								
Yes	230, 35.2%	7.55 ± 5.19	0.005	17, 7.4%	213, 92.6%	0.798	3.79 ± 0.83	0.834
No	423, 64.8%	6.27 ± 5.64		29, 6.9%	394, 93.1%		3.81 ± 0.88	
Community violence								
Yes	527, 80.7%	6.96 ± 5.42	0.020	40, 7.6%	487, 92.4%	0.265	3.82 ± 0.85	0.211
No	126, 19.3%	5.69 ± 5.83		6, 4.8%	120, 95.2%		3.71 ± 0.92	
Collective violence								
Yes	261, 40.0%	8.23 ± 5.65	< 0.001	27, 10.3%	234, 89.7%	0.007	3.85 ± 0.84	0.408
No	392, 60.0%	5.71 ± 5.20		19, 4.8%	373, 95.2%		3.78 ± 0.88	

Exposure to any type of ACEs was associated with a higher mean depression symptom severity score across all ACEs; thus, a history of exposure to at least one ACE was associated with current depression among university students. Individuals who experienced contact sexual abuse, collective violence, and who had incarcerated household member(s) before 18 years experienced suicidal ideations more than those who did not. However, exposure to any type of ACEs wasn’t associated with self-rated academic performance.

### Correlation between the significant variables at inferential statistics

Table 3 represents the correlation coefficients of the statistically significant continuous covariates. There was a low positive significant correlation between depression symptom severity and both the cumulative number of ACEs (*r* = 0.36, *p* < 0.01), and suicidal ideation severity (*r* = 0.46, *p* < 0.01). Therefore, the higher the cumulative number of ACEs reported, the higher the depression symptom severity. Similarly, the higher the depression symptom severity, the more the likelihood of becoming suicidal. (Table 3).

**Table 3:** Correlations between total ACEs with the continuous variables

Study variables	Pearson correlation coefficients (*r*)					
	1	2	3	4	5	6
Age (1)	1.00					
Year of study (2)	0.40**	1.00				
Self-rated academic performance (3)	0.01	− 0.07	1.00			
Depression symptom severity (4)	− 0.22**	− 0.29**	0.13**	1.00		
Suicidal ideation severity (5)	− 0.11**	− 0.13**	0.01	0.46**	1.00	
Cumulative number of ACE (6)	− 0.07	− 0.07	− 0.04	0.36**	0.14	1.00

**p < 0.01. r value represents as; (1) very high correlation (0.90–1.00), (2) high correlation (0.70–0.90), (3) moderate correlation (0.50 to 0.70), (4) low correlation (0.30–0.50), and (5) negligible correlation (0.00–0.30). ACE(s): Adverse Childhood Experience(s)

### Predictive factors for self-rated academic performance and mental health outcomes

In the present multiple regression analysis, all independent variables were assessed for multicollinearity using the Variance Inflation Factor (VIF) analysis. All variables had a VIF < 2 (mean VIF = 1.52), thus were all included in the final adjusted regression model. Three models were generated in multivariate regression analyses to establish associations between the study variables (Table 4).

**Table 4:** Multiple regression models for associations between the cumulative number of ACEs and self-rated academic performance, depression symptom severity, and suicidal ideations

	Self-rated academic performance		Depression symptom severity		Suicidal ideations	
	β (95% CI)	*p* value	β (95% CI)	*p* value	aOR (95% CI)	*p* value
Cumulative No. ACE*	− 0.007 (− 0.031–0.016)	0.558	0.684 (0.531–0.837)	< 0.001	1.264 (1.090–1.465)	0.002
Age*	0.028 (0.001–0.055)	0.043	− 0.312 (− 0.483 to − 0.140)	< 0.001	0.833 (0.594–1.169)	0.291
Gender						
Female	Ref		Ref		Ref	
Male	0.271 (0.140–0.401)	< 0.001	− 1.154 (− 1.988 to − 0.319)	0.007	1.050 (0.441–2.499)	0.912
Marital status						
Single	Ref		Ref		Ref	
Married/cohabiting	0.139 (− 0.155 to 0.434)	0.355	1.340 (− 0.540 to 3.220)	0.162	1	
Separated/divorced	0.429 (− 0.157 to 1.015)	0.151	− 0.547 (− 4.285 to 3.191)	0.774	1.437 (0.093–22.125)	0.795
Region						
Central	Ref		Ref		Ref	
Eastern	0.180 (− 0.013 to 0.372)	0.067	− 1.293 (− 2.521 to − 0.065)	0.039	0.176 (0.039–0.800)	0.025
Northern	− 0.369 (− 0.648 to − 0.090)	0.010	0.211 (− 1.568 to 1.990)	0.816	0.107 (0.008–1.420)	0.090
Western	0.256 (0.099–0.412)	0.001	− 0.713 (− 1.711 to 0.284)	0.161	0.168 (0.054–0.516)	0.002
Type of family						
Extended family	Ref		Ref		Ref	
Grandparent family	− 0.215 (− 0.704 to 0.272)	0.386	4.069 (0.954–7.183)	0.011	2.817 (0.280–28.314)	0.379
Nuclear family	− 0.177 (− 0.324 to − 0.031)	0.018	− 0.052 (− 0.987 to 0.884)	0.913	0.311 (0.118–0.818)	0.018
Single parent family	0.138 (− 0.091 to 0.367)	0.238	− 0.124 (− 1.589 to 1.341)	0.868	0.134 (0.026–0.684)	0.016
Step family	0.019 (− 0.421 to 0.460)	0.933	2.839 (0.028–5.650)	0.048	0.504 (0.060–4.211)	0.527
Primary care provider						
Parent	Ref		Ref		Ref	
Step parent	0.025 (− 0.892 to 0.942)	0.957	2.463 (− 3.390 to 8.315)	0.409	1	
Uncle/Aunt	0.127 (− 0.157 to 0.411)	0.381	1.237 (− 0.576 to 3.050)	0.181	1.117 (0.179–6.963)	0.905
Sibling	− 0.048 (− 0.460 to 0.363)	0.820	1.078 (− 1.548 to 3.704)	0.420	1	
Guardian	0.249 (− 0.043 to 0.542)	0.095	− 0.240 (− 2.108 to 1.627)	0.801	5.021 (1.094–23.047)	0.038
Grandparent	0.136 (− 0.331 to 0.604)	0.568	− 0.654 (− 3.637 to 2.328)	0.667	0.143 (0.017–1.236)	0.077
NGO	0.112 (− 0.249 to 0.473)	0.543	2.515 (0.211–4.818)	0.032	1.123 (0.076–16.658)	0.933
Self-sponsored	− 0.028 (− 0.585 to 0.529)	0.921	5.971 (2.421–9.521)	0.001	8.449 (0.476–149.82)	0.146
No. family members						
0–6	Ref		Ref		Ref	
7–8	0.035 (− 0.160 to 0.230)	0.724	− 0.261 (− 1.503 to 0.980)	0.680	2.140 (0.637–7.191)	0.219
9–10	− 0.038 (− 0.272 to 0.196)	0.748	− 0.205 (− 1.697 to 1.286)	0.787	0.597 (0.116–3.068)	0.537
11–25	− 0.047 (− 0.332 to 0.236)	0.741	− 1.226 (− 3.039 to 0.586)	0.184	2.460 (0.381–15.883)	0.344
No. children in family						
0–4	Ref		Ref		Ref	
5–6	− 0.153 (− 0.347 to 0.041)	0.121	− 0.115 (− 1.354 to 1.123)	0.855	0.428 (0.132–1.391)	0.158
7–8	− 0.164 (− 0.409 to 0.081)	0.189	1.193 (− 0.371 to 2.757)	0.135	1.597 (0.385–6.623)	0.519
9–18	0.133 (− 0.445 to 0.178)	0.400	0.818 (− 1.169 to 2.806)	0.419	0.064 (0.007–0.562)	0.013
Birth position						
1st	Ref		Ref		Ref	
2nd	0.047 (− 0.136 to 0.231)	0.614	− 0.309 (− 1.484 to 0.865)	0.605	0.335 (0.096–1.163)	0.085
3rd	− 0.017 (− 0.201 to 0.166)	0.855	− 0.370 (− 1.543 to 0.803)	0.536	0.341 (0.103–1.130)	0.078
4th and more	0.052 (− 0.128 to 0.232)	0.568	0.195 (− 0.952 to 1.343)	0.739	0.702 (0.239–2.063)	0.520
Mother’s education level						
None	Ref		Ref		Ref	
Primary	0.168 (− 0.060 to 0.397)	0.148	0.939 (− 0.517 to 2.396)	0.206	8.250 (1.602–42.492)	0.012
Secondary	0.279 (0.044–0.514)	0.020	− 0.264 (− 1.763 to 1.235)	0.730	2.518 (0.465–13.628)	0.284
Tertiary	0.336 (0.098–0.575)	0.006	0.337 (− 1.184 to 1.859)	0.663	1.004 (0.183–5.519)	0.997
Father’s education level						
None	Ref		Ref		Ref	
Primary	− 0.003 (− 0.266 to 0.259)	0.980	− 0.614 (− 2.289 to 1.059)	0.471	0.124 (0.017–0.886)	0.037
Secondary	− 0.175 (− 0.425 to 0.075)	0.170	0.076 (− 1.520 to 1.672)	0.925	2.533 (0.534–12.013)	0.242
Tertiary	− 0.191 (− 0.429 to 0.046)	0.113	0.222 (− 1.291 to 1.737)	0.773	2.734 (0.608–12.286)	0.190
CGPA grading						
Third class	Ref		Ref		Ref	
Second class—lower	0.220 (− 0.212 to 0.653)	0.317	− 1.853 (− 4.609 to 0.903)	0.187	0.192 (0.022–1.708)	0.139
Second class—upper	0.948 (0.521–1.374)	< 0.001	− 2.647 (− 5.366 to 0.072)	0.056	0.034 (0.003–0.299)	0.002
First class	1.533 (1.085–1.981)	< 0.001	− 2.546 (− 5.402 to 0.309)	0.080	0.094 (0.010–0.891)	0.039
Preferred not reporting	0.796 (0.352–1.242)	< 0.001	− 1.810 (− 4.648 to 1.029)	0.211	0.136 (0.015–1.204)	0.073
Year of study*	0.083 (0.013–0.153)	0.019	− 0.598 (− 1.043 to − 0.153)	0.009	1.182 (0.630–2.219)	0.603
Faculty of study (17)						
Applied sciences and technology	Ref		Ref		Ref	
Business and management studies	− 0.073 (− 0.180 to 0.326)	0.571	1.282 (− 0.330 to 2.894)	0.119	0.815 (0.210–3.169)	0.768
Computing & informatics	0.350 (0.073–0.626)	0.013	0.607 (− 1.155 to 2.370)	0.499	0.461 (0.100–2.119)	0.320
Interdisciplinary studies	0.135 (− 0.410 to 0.681)	0.627	− 0.829 (− 4.309 to 2.651)	0.640	1	
Medicine	− 0.042 (− 0.267 to 0.181)	0.710	− 0.923 (− 2.355 to 0.509)	0.206	0.052 (0.011–0.237)	< 0.001
Science	0.209 (− 0.031 to 0.450)	0.088	0.442 (− 1.091 to 1.975)	0.572	0.559 (0.140–2.237)	0.411
Constant	1.851 (1.057–2.645)	< 0.001	14.117 (9.051–19.183)	< 0.001	79.005 (0.109–57,308.87)	0.194
Observations	653		653		589	
p values	< 0.001		< 0.001		< 0.001	
R^2^	0.3108		0.3107		0.2912	

No. = Number of; FmW = Family members with; ACE(s) = Adverse Childhood Experience(s); β = Adjusted beta coefficient, CI = Confidence Intervals; NGO = Non-Governmental Organization; CGPA = Cumulative Grade Point Average

*Continuous variables

In model 1, after adjusting for other independent variables, the cumulative number of ACEs reported were not associated with self-rated academic performance (β =  − 0.007; 95% CI − 0.031 to 0.016; *p* = 0.558). However, increase in age, male gender, residing in the western region of the country, mothers having a secondary level or tertiary education level as the highest attained education level, having a current CGPA grading of the second class—upper or first class, as well as those who preferred not to report their CGPA, year of study, and belonging to the facility of Computing and Informatics, were significantly positively associated with self-rated academic performance. Conversely, negative associations were with coming from the Northern region of the country and from a nuclear type of family.

In Model 2, after adjusting for all other variables, the cumulative number of ACEs reported was positively associated with depressive symptom severity (β = 0.684; 95% CI 0.531–0.837; *p* < 0.001), showing 0.684 increase in the depression symptomatology score, for every additional ACE reported. In addition, living with a grandparent or step-family, and being sponsored by an NGO or self-sponsorship were positively associated with the depression symptom score. However, the increase in age, male gender, originating from the country's Eastern region, and year of study were negatively associated with depression symptom severity.

In Model 3, a history of exposure to ACEs was associated with 1.264 times increase in the likelihood of developing current suicidal ideation (aOR = 1.264; 95% CI 1.090–1.465; *p* = 0.002), after adjusting for all other studied predictor variables. In addition, having a guardian, as the primary care provider and mothers with primary education level increased the likelihood of having suicidal ideations. However, originating from the Eastern or Western regions of the country, coming from nuclear family or single parent family, a family with nine to 18 children, father’s highest education level being primary education, current CGPA grading of second class—upper or first class, and belonging to the faculty of medicine significantly reduced the likelihood of having suicidal ideations.

Moreover, predictor variables for all these three statistically significant models explain about a similar variance of about 30%.

## Discussion

The present study is the first of its kind in Uganda to be conducted among university students investigating exposure to adverse childhood experiences (ACEs), family environment, and possible associations with self-rated academic performance, depressive symptoms, and suicidal ideation. This study gathers evidence of an overwhelmingly high burden of ACEs among university students, highlighting the importance of child protection in the country.

Almost all (99.8%) study participants reported exposure to at least one of the 13 ACEs assessed in this study. Such a high level of multi-type child maltreatment exposure (97.6%) was reported in a study conducted among Tanzanian college students [12]. However, most of the available studies among university students from other countries reported a lower prevalence of experiencing at least one ACE, than that reported in this study; for example, Botswana (73%) [6], Tunisia (74.8%) [56], and Zambia (58.3%) [11]. It should be noted that the reasons for intrinsic variations in the prevalence of experiencing at least one ACE within LMICs may lie in tools used to assess the ACEs. Moreover, this study investigated 13 types of childhood adversities using the ACE-IQ tool [49], compared to the vast majority of studies that measure less than 13 ACEs [6, 11, 56]. Measuring more ACEs may, on the one hand, enhance ecological and cross-cultural validity, but on the other hand, it may make the comparability of data with previous studies difficult [57]. This study is consistent with many national and international studies that show that various childhood adversities co-exist [6, 7, 9, 10, 56, 58], with this study reporting over 70% of participants experiencing five or more childhood adversities. This is sufficient evidence that children in Uganda are often subjected to poly-victimization, and a growing body of literature, confirms that most worrisome outcomes are associated with high multiple exposures of ACEs [6, 11, 19].

The commonest types of ACEs reported by students in this study were physical abuse, emotional abuse, emotional neglect, community violence, and household dysfunction (99.8%, 57.3%, 68.0%, 80.7%, and 66.3%, respectively). This result is consistent among university students in the eight provinces of Vietnam, who commonly reported emotional abuse, physical abuse, and witnessing a household member being treated violently (42.3%, 39.9%, and 34.6%, respectively) assessed using the same ACE-IQ screening tool [16]. In another study among social work students of California State University in the USA, the frequently reported ACEs included physical abuse and emotional neglect [59]. It is noted that the majority of studies in Africa point out physical abuse as one of the frequent ACEs reported [6]. This agrees with this study, where physical abuse was the main form of abuse reported among this student sample with only one participant who wasn’t physically abused. Therefore, the findings of this study demonstrate that growing up in a violent and pugnacious environment is relatively common among children in Uganda. This is due to the in-built African cultural and behavioral values and beliefs, such as patriarchal norms, endorsement of physical punishment, and normalization of family violence, which exposes children to physical abuse [60]. Although children need to be nurtured and protected by adults, children may experience inappropriate punishment strategies that do not promote behavioral self-regulation or self-discipline. Instead, this may lead to an adverse change of behavior due to fear and not realizing their wrongs, which self-perpetuates into violence and victimization later in their adulthood [61]. This should persuade political decision-makers to set priorities and approve legal child protection policies to promote safe child upbringing in this multicultural group [62]. However, with the ‘yes’ or ‘no’ responses for the ACE-IQ (i.e., *Item 1: Did a parent, guardian or other household member spank, slap, kick, punch or beat you up? Item 2: Did a parent, guardian or other household member hit or cut you with an object, such as a stick (or cane), bottle, club, knife, whip *etc*.?*), which does not put into consideration the severity of the physical abuse experienced, distinguishing between corporal and other loving/out of care punishments is difficult. Besides, in Uganda corporal punishment as form of discipline is common both in schools and home environments [63, 64]

Previous studies among young adults report that child maltreatment negatively affects academic performance in college students [24, 65]. For example, US middle and high school students showed that more intense childhood maltreatment are associated with a lower grade point average and problems in completing homework assignments among college adolescents [26]. Similarly, an association between ACEs exposure and post-secondary academic performance among African-American college students in the USA, was reported in a recent study, where higher ACEs scores were associated with lower grade point averages [24]. In Uganda, academic performance among undergraduates has been associated with student factors (like grades in the previous school), parental socioeconomics, and the presence of students’ set goals [66]. Although this study explored the possible association between academic performance and ACEs for the first time, no significant association was found (see Tables 2, 4). This suggests that factors other than ACEs exposure, may have confounded the negative impact of ACEs. These factors may include low socioeconomic status, relationship problems with teachers and peers, poor living arrangements, risky behavioral patterns, and rural–urban differences, which were not assessed for in this study [7, 9, 57].

In this study, a higher cumulative number of ACEs experienced was positively associated with a current higher depression symptom severity during adulthood, a finding consistent with prior studies in sub-Saharan Africa [3, 6, 9] and elsewhere [1, 4, 12]. This result is consistent with findings in a USA national children and adolescent sample, which concluded that psychological maltreatment is associated with increased odds of depression later in life [10, 67]. Approximately 280 million people suffer from depression worldwide, and people from low- and middle-income countries like Uganda are more vulnerable since more than 75% of them receive no treatment or interventions [68]. Interventions aiming at reducing ACEs such as child protection services, and educating the population about the dangers of ACEs, among others may reduce ACEs and thus reduce the burden of depression.

Approximately 90% of suicide cases are attributed to at least one mental disorder, and depression is listed in most of these cases [69, 70]. Similarly, an increase in the cumulative number of ACEs causes a 1.264 increase in the likelihood of developing suicidal ideation. This finding is consistent with the prior studies that show that persons exposed to ACEs become suicidal in their later life [10, 67, 71]. In a sample of Ugandan youths residing in urban areas, experiencing physical or emotional abuse and neglect was found to result in over two times increase in their odds of developing suicidal ideation later in life [72]. However, suicidality among university students has multifactorial causes (not only exposure to ACEs) such as relationship problems, gambling, scams, and family misunderstandings [73–75]. Amongst other factors, early recognition of childhood adversities and employing appropriate measures to address them is essential in avoiding mental health issues like depression, suicidality, etc., throughout the life span of an ACEs victim.

### Limitations

This exploratory study has limitations to be noted. First, the study lacks causal inference due to the cross-sectional nature of the study design. Although Springer et al. [76] argue that the causal association is met in retrospective ACEs studies, we consider this a limitation due to the potential bias associated with retrospective self-report measures in cross-sectional studies such as recall bias since individuals were required to report what transpired at < 18 years. Secondly, this study faced bias, especially selection bias due to disproportional faculty representation, and social desirability bias, especially in self-rated academic performance (although attempts were made to assume anonymity). Thirdly, the study was conducted during the COVID-19 pandemic which was characterized by high rates of mental illness, especially, among university students [77–80], which could have affected the mental health symptoms of the student. Fourth, the predictability of the models were low (i.e., approximately 30%), meaning other factors may be responsible for the mental health symptoms and academic symptoms. We recommend future studies to explore other factors to have better explanation and understanding of student’s mental health problems. Lastly, the study considered parents’ highest level of education even for orphans (this study did not ask for information about parental death since this could be an important information). However, parents’ education may still play a significant role in the adult experience for individuals. Future studies should explore this aspect and determine the influence of parents’ level of education on ACEs, mental health symptoms, or academic performance.

Notwithstanding the study's shortcomings, the analysis provides a deeper insight into the burden of ACEs among university students in Uganda, although this community is considered exceptionally privileged. The association and correlations with the family environment highlight that the starting point in preventing child exposure to ACE is the basic family unit. Poor academic performance and mental health problems (i.e., depression, suicidal ideation) might have resulted from high ACEs exposure.

### Implications to research, policy, and practice

Public health efforts should focus on preventing ACEs; designing, testing, and implementing interventions to increase capacity for children and adults. Regulatory policies need to be strengthened to ensure a healthy environment throughout the life cycle. Future research should standardize culturally appropriate tools to explore the different forms of childhood abuse experienced locally and qualitatively assess adaptive strategies used by ACEs victims to develop resilience. In addition, other factors that may influence the mental health of ACEs victims should be assessed for implementers to develop targeted interventions.

## Conclusions

The burden of ACEs is unacceptably high among Ugandan university students, highlighting the urgency of establishing and strengthening effective child protection strategies in the country. With the family environment being the highest predictor, ACEs prevention projects should target families as basic community units in a collective effort to create a safe atmosphere for child development. This may reduce complications, awful ACEs-associated mental health challenges, and poor academic performance among university students later in life.

## Data Availability

The dataset used and/or analyzed during the current study are available from the corresponding author on reasonable request.
